# Effect of body position on relieve of foreign body from the airway†

**DOI:** 10.3934/publichealth.2019.2.154

**Published:** 2019-04-24

**Authors:** Artur Luczak

**Affiliations:** Canadian Centre for Behavioural Neuroscience, Department of Neuroscience, University of Lethbridge, 4401 University Drive, Lethbridge, AB, T1K 3M4, Canada

## Abstract

Foreign body airway obstruction (FBAO), or commonly known as choking, is an extremely dangerous event. The European Resuscitation Council recommends that back blows and abdominal thrusts should be performed for relieving FBAO in conscious adults. Reviewed here evidence suggests that applying a prone or a head-down position increases effectiveness of the above standard approaches to relieve obstruction, due to help of gravity.

## 1. Introduction

Choking is an emergency situation, and it can quickly result in death if not treated promptly [1]. The lack of oxygen caused by choking can result in brain damage or death in four to six minutes. Unless immediate action is taken to open a completely obstructed airway, the chances for survival and complete recovery decrease rapidly [2]. It is estimated that choking can result in tens of thousands of deaths each year (incidence of 0.65–0.9:100,000) [3],[4]. However, this may be a gross underestimation, as many additional deaths due to choking could be mistakenly ascribed to other causes, such as myocardial infarction [5].

For conscious adults showing signs of severe airway obstruction, the European Resuscitation Council recommends applying back blows followed by abdominal thrusts [6]. Unfortunately, in some cases, these techniques were reported to cause serious complications. For example, back blows may lodge the foreign object more tightly in the trachea [7], and abdominal thrusts (Heimlich maneuver) may cause the rupture of internal organs [8],[9]. Similarly, chest compressions are known to cause rib and sternal fractures; although usually benign, these fractures can lead to additional morbidity such as pneumonia or even death from respiratory insufficiency [1].

Despite the obvious need to develop safer approaches for FBAO treatment, and to investigate how to reduce complications following the application of the above maneuvers, there is little research which could guide the best treatment for choking[10]. One of the main reasons for the lack of studies with proper controls on FBAO treatment is obvious ethical concern. For that, the analysis of efficacy of various therapeutic techniques must rely heavily on case studies based on isolated individuals [11].

One other important limitation of current recommendations for FBAO treatment is that it requires the assistance of another person, and in approximately 30–40% of FBAO cases, the victim is alone [12],[13]. Surprisingly, even if other people are present during a FBAO, in only 5–13% of these cases are the observers or medical personnel able to make a correct diagnosis and initiate the appropriate treatment [12],[13]. These statistics underscore the importance of developing an alternative method of treatment which could be self-applied without relying on the presence or knowledge of other people.

## 2. Effect of body position

**Figure 1.**
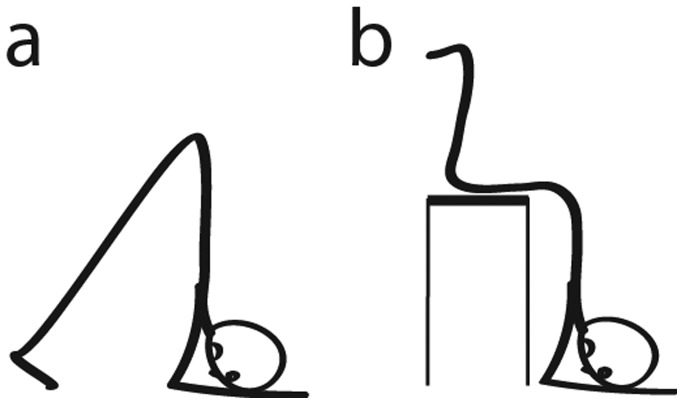
Head-down position to prevent suffocation in choking. (a) Simple initial position. (b) Position with the help of a stool (figure reproduced with permission from [28]).

One of the approaches to improve FBAO treatment methods could be by taking advantage of help of gravity. Below are reviewed evidence strongly suggesting that combining any of the above treatment methods with a prone or head-down (inversed) position (Figure 1) could be more effective at dislodging an obstructing object than any of the treatments alone. The head-down position is already recommended during choking incidents that occur in children below one year of age. It is advised that in infants, back blows should be applied with the head downward to enable gravity to assist with the removal of a foreign object [6],[14],[15]. Similarly, it is suggested that in older children that back blows are more effective if the child is positioned head down [15]. There is also evidence that combining the inverse position with other treatment methods improves its effectiveness. For example, Briole [16] reported that of the four Heimlich procedures performed on children, three of the procedures were effective when performed by placing the child upside down (head-chest down), while the one procedure performed with the child laying down was ineffective. There are also multiple case studies showing that using the inversed position method in children facilitated successful treatment of FBAO. In one such case study, the removal of a laryngeal foreign body was accomplished by a physician only when using a laryngoscope and finger sweep was combined with lifting a six year-old boy upside down by his feet [17]. In another case study, where traditional methods of FBAO treatment have failed, by keeping a 12-month-old girl with her head lower than her buttocks in a prone position, and with the help of mouth-to-mouth aspiration, obstructing object was successfully removed[18]. It has also been reported that “head-down shaking” was a successful treatment method for foreign body extraction in pediatric patient [19].

Considering that the head-down position is already recommended for children, and even for choking pets [20], it is surprising that for adults similar recommendations are not included in resuscitation guidelines. Only in very few guidelines it is recommended to lean the adult victim well forward during back blows [6] which may result in a partial head-down position. One of the reasons for the lack of recommendations for using the inverse position in adults could be that the literature characterizing the occurrence of FBAO incidents is considerably smaller in case of adults than in children [21], as choking is most likely to occur in children below two years old of age [22]. Nevertheless, available evidence reviewed below strongly suggest that there is substantial support to recommend a prone or head-down position also in adults.

For example, the effectiveness of the prone/head-down position was reported in four cases of choking in unconscious elderly patients. After failure of the Heimlich maneuver, the choking person was laid down on the table in the prone position with the head and arms of the patient hanging downwards beyond the table. Then back blows were given which resulted in dislodging the foreign body [23].

One of the earliest most rigorous studies of different methods for foreign body removal was conducted by Ruben and Macnaughton [24]. They studied FBAO in artificial larynxes attached to the endotracheal tubes of intubated volunteers, and systematically compared the effectiveness of chest thrusts, abdominal thrusts, and back blows. They found that pressures required to remove a piece of food wedged in the larynx, in an airtight position, was far higher than those which could be achieved with any of the treatment methods. They reported that successful removal of the obstructing food could be achieved only when maneuvers were aided by gravity. Thus, they emphasize the positive effects of inverting the patient. Similar results were obtained recently using a Heimlich maneuver on a mankind [25]. It was showed that a prone position was the most effective at relieving a supralaryngeal obstruction. In another mankind study, abdominal thrust were also more effective when mankind was in laying down vs standing position [26]. Those results are consistent with experiments on anaesthetised pigs, where thoracic thrusts generated higher airway pressure in laying down (lateral) position as comparing to upright position [27].

Moreover, I would like to mention that I also experienced food choking, and using myself the procedure of the upside-down position helped me to remove the object from my airway, which later prompted me to investigate this procedure as an additional method to alleviate choking [28]. Personal experience account of medical emergency is quite rare, however it can provide additional perspective. For example in my case, saliva entering trachea was particularly distressing during FBAO accident. Thus it is surprising that effect of saliva is usually not considered in FBAO literature. Notably, one other advantage of the head-down position is that it could help remove saliva and other fluids which may further obstruct airflow during choking; particularly during partial obstruction or when a foreign object is semisolid. This procedure is similar to postural drainage when using an inverted position in patients with chronic inflammation in airways, as it helps remove fluids with the aid of gravity [29],[30]. For example, in a case study [18] the head-down position helped to remove saliva which was an additional factor obstructing airways during a FBAO.

## 3. Potential concerns

The main concern for head-down position is that when an object is lodged below the glottis (vocal cords), then inversion may cause a complete obstruction when the object falls on the vocal cords. Nevertheless, the infraglottic obstruction accounts for only about 25% of cases [13]. Therefore in majority of FBAO cases inversion should be a viable option, especially when applied as last resort method to minimize this concern. Another potential concern about the head-down position procedure is that it could reduce the effectiveness of a natural cough. Unfortunately, there are no studies that directly measure the strength of a cough in the head-down vs upright position. However in related studies, side lying in a prone position was shown to be as effective for coughing up secretions as upright sitting positions [31], and gravity-assisted positioning was reported to be more effective than cough alone in subjects with excessive bronchial secretions [30],[32]. Nevertheless, considering scarcity of data on cough efficiency in the head-down position, the safest option could be to apply the inversed position as the last option if all other maneuvers fail. Further studies on this subject would be beneficial.

## 4. Conclusions

Review of available literature suggests that a prone or a head-down position could alleviate a foreign object obstruction of airflow by the aid of gravity. Thus this position could be of great benefit to a choking victim, especially when other treatment methods fail. For these reasons, it comes as a surprise that the prone or head-down position method is not mentioned for treating adults suffering from a FBAO. The upside-down body position is generally safe, and it is commonly used in variety of exercises. Research also shows that while having a choking person in a prone or a head-down position, the effectiveness of chest thrusts and back blows could be enhanced due to the gravitational pull on the obstructing object [5],[23],[24]. In summary, the data presented here suggests that the head-down position could be an effective method of rescuing adults suffering from a FBAO when other methods are not successful or not available (especially when a choking person is alone), thus potentially saving lives.
